# Perceived occupational stress and associated factors among primary school teachers in the second wave of COVID-19 in Ethiopia: a multicenter cross-sectional survey

**DOI:** 10.3389/fpubh.2023.1156652

**Published:** 2023-06-19

**Authors:** Amensisa Hailu Tesfaye, Kassahun Abate, Gebisa Guyasa Kabito, Telake Azale

**Affiliations:** ^1^Department of Environmental and Occupational Health and Safety, Institute of Public Health, College of Medicine and Health Sciences, University of Gondar, Gondar, Ethiopia; ^2^West Wellega Zone Labor and Social Affairs Department, Nekemte, Ethiopia; ^3^Department of Health Education and Behavioral Sciences, College of Medicine and Health Sciences, University of Gondar, Gondar, Ethiopia

**Keywords:** occupational stress, primary school, teachers, COVID-19, Ethiopia

## Abstract

**Background:**

Occupational stress (OS) is a psychological state that results from people’s perceptions of an imbalance between job demands and their abilities to cope with those demands. The COVID-19 pandemic has disrupted the teaching and learning process and compounded the stress level among teachers due to fear of transmission of the virus and school closures or the difficulty with adherence to the COVOD-19 prevention protocol. This survey study therefore aimed to investigate the prevalence of occupational stress and its associated factors among primary school teachers during the second wave of COVID-19 in western Ethiopia.

**Method:**

An institution-based cross-sectional survey was employed from April to May 2021. The survey was conducted in Gimbi town among all 672 primary school teachers in western Ethiopia. The standardized Teacher Occupational Stress Scale was used to measure occupational stress in the past 4 months. The data were collected through a self-administered questionnaire. The collected data were entered into EpiData version 4.6 and analyzed using Stata version 14 software. A multivariable logistic regression analysis was conducted to identify factors associated with occupational stress. The statistical significance was considered at a value of *p* of < 0.05 and a 95% confidence interval (CI) with an adjusted odds ratio (AOR) to evaluate the strength of associations.

**Results:**

The response rate was 96.8% (*N* = 651). The majority, 389 (59.8%) of the study participants were males. The mean (±SD) age was 35.8 (±9.3) years. The prevalence of occupational stress in the second wave of COVID-19 in the past 4 months was 50.1% (*n* = 326) [95% CI (46.1, 53.9)]. Job dissatisfaction [AOR: 2.06, 95% CI (1.43–2.97)] and high-risk perception of COVID-19 infection [AOR: 2.20; 95% CI (1.46–3.31)] were significantly associated with occupational stress.

**Conclusion:**

This survey disclosed a high prevalence of occupational stress among primary school teachers during the second wave of COVID-19. Job dissatisfaction and a high-risk perception of COVID-19 infection were significant predictors of the occurrence of occupational stress in school teachers. Enhancing stress management skills and focusing on primary prevention of identified risk factors were advised to curtail the condition.

## Background

Occupational stress (OS) is a psychological state that results from people’s perceptions of an imbalance between job demands and their abilities to cope with those demands (1). Occupational stress emerged when workers tried to cope with tasks, responsibilities, or other forms of pressure connected with their jobs but encountered difficulty, strain, anxiety, and worry (2). Teaching has been confirmed as one of the most stressful, strenuous, and difficult occupations, with a higher annual turnover rate than other professions (3, 4). Particularly, teaching in primary schools is more challenging and demanding (5), and the occurrence of OS is exacerbated by the emergency of the COVID-19 pandemic. Moreover, the pandemic has disrupted the teaching and learning process and compounded the stress level among teachers due to fear of transmission of the virus and school closures or the difficulty with adherence to the COVID-19 prevention protocol (6).

As a result of the COVID-19 pandemic, school closures have affected the education of many students around the world (7). Schools in Ethiopia were closed in March 2020 as the pandemic spread and did not reopen for face-to-face education until October 2020 (8). Schools in all countries returning to face-to-face teaching have taken safety measures to reduce the risk of spreading COVID-19, such as increased ventilation, social distancing, body temperature control, and wearing masks and shields. Adapting to COVID-19 safety protocols presented challenges for teachers (9, 10). For example, masking muffles the voice and makes it difficult for students and teachers to recognize facial expressions. Due to increased interactions with students and peers during face-to-face instruction, teachers also indicated that they were concerned about their health and the health of their families and communities (11). The coronavirus pandemic has placed significant stress on the teaching profession, even in schools that have reopened to face-to-face teaching (9, 12).

Thus far, the number of cases of OS has continued to increase over the years in every occupation, predominantly among employees involved in the educational sector, including elementary school teachers (13). Before the COVID-19 pandemic, the rate of self-reported occupational stress had been increasing, and the rate is higher than the 2018/2019 pre-coronavirus levels (14). According to the most recent Labor Force Survey (LFS) estimates, the total number of cases of occupational stress, depression, or anxiety in 2021/2022 was 914,000, a prevalence rate of 2,750 per 100,000 workers (13). The pooled prevalence of OS among teachers of different levels during the COVID-19 pandemic is reported to be 30% globally (15). There are wide variations in rates of OS among teachers as reported in different countries. In the United Kingdom, 2,100 out of every 100,000 educators suffer from OS (16), 45% in Ireland (17), 42% in Greece (18), 33.2% in Kosovo (19), 65% in India (20), 44.9% in Pakistan (21), 34% in Malaysia (22), 31.1% in South Africa (23), 100% in Egypt (24), and 58.2% in Ethiopia (25).

As teachers are responsible for producing the major task force of any country, their psychological health and performance have an important impact on the economy (5). The total estimated cost of occupational stress is estimated to range from US $ 221.13 million to $ 187 billion in most European countries, $221 million and to $580 million per year in Australia, $187 billion in the United States (26), and the cost of the problem is continuing to upsurges in developing countries despite the extent of the issue is uncertain due to a lack of statistics (27).

On top of that, OS is one of the major public and occupational health problems that lead to various symptoms such as anxiety, headaches, sleep disturbances, and irritability (28), as well as depression, frustration, hypertension, suicidal thoughts, and attempts (23), gastric illness, and cardiovascular manifestations (18, 23). From an occupational health perspective, OS leads to chronic job absenteeism, poor performance, and high teacher turnover (29). According to literature from North Carolina, around 28% of teachers left the profession (30). In the Ethiopian context, about two-thirds of teachers planned to resign from their teaching occupation (31). Existing studies confirm that the risk of depression increases up to 4 times, among workers experiencing OS (32).

The number of factors that are most frequently reported as contributing to OS are bad social climate, workload, work demands (33), inadequate wages, physical working environment, undisciplined students (19), class size, time, professional investment, job dissatisfaction, role overload, poor school management, insufficient resources, teachers’ low social status, a lack of parental support, a lack of support from coworkers, a lack of job stability and security, a lack of career development (23, 34), job changes (33), and taking home work (28).

Even though the existing evidence on occupational stress is predominantly from developed countries. However, little is known about the prevalence and determinants of occupational stress among primary school teachers in sub-Saharan Africa, including Ethiopia, during the period of the COVID-19 pandemic. Therefore, this study aimed to investigate the prevalence of occupational stress and its associated factors among primary school teachers during the second wave of COVID-19 in Gimbi town, western Ethiopia.

## Materials and methods

### Study design, period, and setting

A cross-sectional survey was employed during the second wave of COVID-19 in western Ethiopia from April to May 2021. This study was conducted on public and private primary school teachers in Gimbi town. The town is located in the West Wollega Zone of the Oromia Regional State, 440 km in the west direction of Addis Ababa, Ethiopia. There were 18 public and 5 private primary schools in and around the town.

### Study populations

All primary school teachers (grades 1–8) in Gimbi town were the study population. Primary school teachers who had at least 1 year of working experience were eligible for this study (35), while school teachers with a history of psychiatric problems and those on annual, sick, family, or maternity leave were excluded from the study.

### Sample size determination and sampling procedure

We used Epi Info version 7 software to calculate the sample size required for the study using the following assumptions: 95% CI, 80% power, 1:1 ratio, AOR 1.61, % outcome of the unexposed group: 52, 10% non-response rate, and using factors significantly associated with occupational stress among teachers, which were reported from secondary school teachers in Ethiopia (25). This gave a maximum sample size of 666. However, all the primary school teachers in the town were surveyed due to their small number, which contributes to the strength of the study’s conclusions, and all the teachers in the town can be approached (feasible for data collection). During the study period, there were a total of 672 primary school teachers working in all the primary schools in Gimbi town.

### Operational definitions

Occupational stress: A total score of 50 or higher on the Teachers’ Occupational Stress Scale (TOSS) was classified as the presence of OS, while a score below 50 was classified as the absence of occupational stress (36, 37).

Primary school teachers: Teachers who educate grades 1 to 8.

Job demand: Is measured using 8 items of the HSE tool and categorized as low or high based on the mean value, with a teacher’s score below the mean value classified as low job demand (38).

Role ambiguity: Is measured using 5 items of the HSE tool and categorized as low or high based on the mean value, with a score below the mean value classified as low role ambiguity (38).

Colleague relationships: Teachers who scored at or above the mean were classified as having poor colleague relationships, while teachers who scored below the mean value were classified as having good colleague relationships using the four items of the HSE tool (38).

Managerial support: Teachers who scored at or above the mean were classified as having poor managerial support, and those who scored below the mean had good managerial support using the five items of the HSE tool (38).

Peer support: Using the four items of the HSE tool, teachers who scored at or above the mean were classified as having low peer support, while those who scored below the mean were classified as having high peer support (38).

Job control: Teachers who scored mean and above were categorized as low job control and high job control if scored below the mean value using the six items of the HSE tool (38).

Job change: Teachers who scored mean and above were categorized as poor job change and good job change if scored below the mean value using the six items of the HSE tool (38).

Perceived school physical work environment: Is measured using NIOSH generic job stress questionnaires and dichotomized as good or poor based on the average value, in which average and above the score value is classified as a good physical work environment (39).

Risk perception of COVID-19 infection: Was assessed by two psychological dimensions; perceived susceptibility and perceived severity. The first dimension was proxied by how likely one considered oneself (his/her family) would be infected with COVID-19 if no preventive measures will be taken. The second dimension was proxied by how one rated the seriousness of symptoms caused by COVID-19, their perceived chance of having COVID-19 cured, and that of survival if infected with COVID-19. By combining the two dimensions, five items with five response options were asked to determine the respondents’ levels of risk perception, with a higher total score indicating a high perceived risk of COVID-19 infection (40).

Perceived job satisfaction: A total score of at least 32 on the general job satisfaction scale (41).

Doing physical exercise: Exercising or doing any kind of sports activity at least two times per week with a duration of at least 30 min (42, 43).

Cigarette smoking: It is the practice of smoking a cigarette by teachers for at least one stick of cigarette per day (44).

Alcohol drinking: The consumption of any type of alcoholic-based beverage, whether locally or industrially produced, by teachers in any quantity at least twice per week (44).

Khat chewing: Chewing khat in any form or volume three times a week for at least 12 months (45, 46). Khat is a plant whose leaves are chewed or dried, crushed, and drunk as a tea in the East African region, as well as some parts of the Middle East, for stimulation or enlightenment (47, 48).

### Data collection tools and procedures

Data were collected by using a self-administered and structured questionnaire. A questionnaire was adapted after investigators reviewed related studies (9, 36, 38, 41, 49, 50). The questionnaire was originally designed in English and then translated into Amharic, the local language of the study area. Trained data collectors administered the questionnaire to study participants at their workplaces and collected completed surveys from each participant in person (thought face to face). The standardized Teachers` Occupational Stress Scale (TOSS) was used to measure the proportion of OS among study participants (36). The scale comprised 20 negatively worded questions ranging from 1 to 5 on each item, and the responses ranged from strongly agree = 5, agree = 4, undecided = 3, disagree = 2, and strongly disagree = 1. Based on the scoring system of the tool, we summed up all the scores out of 100, and we dichotomized the scores into a score of less than 50 = 0 (no stress) and a score of 50 and above = 1 (stress) (36).

We evaluated organizational factors with the Health and Safety Executive’s management standards indicator tool, which has standardized 35 items with a five Likert scale of measurement (Never = 1, Seldom = 2, Sometimes = 3, Often = 4, and Always = 5), grouped into demand (8 items), job control (6 items), management support (5 items), peer support (5 items), job relationships (4 items), role ambiguity (5 items), and job change (3 items) (49). According to other studies, we calculated the mean for each group to obtain categories of responses (49).

Risk perception regarding COVID-19 was measured using two psychological dimensions: perceived susceptibility and perceived severity. The 1st dimension (perceived susceptibility) includes two questions: how likely they believe they will be infected with COVID-19 and how likely they believe they or their family will be infected with COVID-19 if no preventive measures are taken. Responses to the two questions were rated on a 5-point Likert scale ranging from 1 = very likely to 5 = very unlikely. The 2nd dimension (perceived severity) contains three questions, including how one rate the seriousness of symptoms caused by COVID-19, their perceived chance of having COVID-19 cured, and their chance of survival if infected with COVID-19. Responses to the three questions were rated on a 5-point Likert scale ranging from 1 = “very serious” or “very low” to 5 = “not serious at all” or “very high.” By combining the two dimensions, five questions are each answered on a Likert scale of 1 to 5, giving rise to a total score ranging from 5 to 25. The higher the score, the higher the perceived risk of infection with COVID-19 (50).

We evaluated the perceived satisfaction of teachers with their jobs using the 10-item generic job satisfaction scale questionnaire. This instrument is a 5-point Likert scale with responses ranging from 1 to 5 (strongly disagree = 1, disagree = 2, neutral = 3, agree = 4, and strongly agree = 5). The response categories were totaled and summed out of 50. The total work-related scale was divided into two categories: a score of less than 32 = 0 (dissatisfied), and a score of 32 and above = 1 (satisfied) with their current jobs (51). We used one section of the National Institute for Occupational Safety and Health (NIOSH) standardized generic job stress questionnaire to assess perceptions regarding the schools’ physical work environment by using 10 items with a reverse score of (1, 2, 5, 9, 10), in which a score of average or above is considered good and a score below average is considered poor (52). Data on basic socio-demographic characteristics, like sex, age, educational status, marital status, monthly salary, work experience, and family size, were also collected. Furthermore, behavioral factors like alcohol use, cigarette smoking, khat chewing, sleep time, and sleep disturbance were assessed.

### Data quality assurance

To ensure the quality of the data collected, we gave much emphasis to the appropriate design of data collection tools. The questionnaire was first developed in English and translated into the local language, Afan Oromo, and back to English by language experts and professionals to ensure consistency. Second, we employed two data collectors and one supervisor with prior experience and knowledge of the data collection process. The data collectors and supervisor took 2 days of training and orientation before the actual data collection on issues relating to the clarity of the questions, objectives of the study, confidentiality of information, informed consent, as well as the roles and responsibilities maintained by both the data collectors and supervisor during the data collection process. The principal investigator supervised both data collectors and supervisors. Third, we conducted a pretest 1 week before the actual data collection period on 5% (34) of the sample size in the Oromia regional state in Nedjo town nearby Gimbi town, which helped us test the validity and consistency of the instrument used. The pretested questionnaires were not included in the study’s analysis. Based on the findings from the pretest analysis, we modified some words and misinterpretations, minimized the number of questions, and made corrections to some other ambiguities. Problems faced during the data collection process were solved by a discussion on the spot with the principal investigator, supervisor, and data collectors.

### Data management and statistical analysis

Data were coded, labeled, verified, categorized, and entered into EpiData version 4.6 software. We used Stata version 14 software to analyze the entered data and computed frequencies, percentages, means, and standard deviations to present the findings. Before doing bivariable and multivariable binary logistic regression analyses, the variables’ normality, outliers, and multicollinearity were examined. A variance inflation factor (VIF) was used to test the multicollinearity assumption, and all variables displayed values of less than five. As a result, we found no evidence of multicollinearity. The reliability of the standardized Teachers’ Occupational Stress Scale was tested using Cronbach’s alpha, which was found to be 0.85. According to Cronbach’s alpha, the reliability of an instrument is tolerable at a cutoff point of 0.65 and above (37, 53, 54). Each dimension of HSE was also examined for reliability, and Cronbach’s alpha values were 0.85 for job demand, 0.79 for role ambiguity, 0.86 for colleague relationship, 0.814 for managerial support, 0.82 for peer support, 0.87 for job control, 0.83 for a job change. The 10-item job satisfaction scale questionnaire was also examined for its reliability, and Cronbach’s alpha was found to be 0.786. The instruments were, therefore, tolerable for their consistency in repeating what had previously been measured using these tools. A binary logistic regression analysis was performed separately for each independent variable to explore the associations with the dependent variable (occupational stress). Significant predictors with a value of p of <0.2 in the bivariable logistic regression analysis were exported to the multivariable logistic regression model using the backward conditional variable selection method to control the potential effects of confounders. We checked the goodness of fit model using the Hosmer-Lemeshow test, and we found the model was a good fit (value of *p* = 0.352) (55). Lastly, a statistically significant association was declared at a cut-off <0.05 value of p to evaluate the significance and an adjusted odds ratio (AOR) with 95% confidence intervals (CI) to establish the strength of associations in the multivariable logistic regression model.

## Results

### Socio-demographic characteristics of the respondents

The response rate was 96.8%. Of those surveyed, 389 (59.8%) were men. The respondents’ ages range from 21 to 70, with a mean (±SD) of 35.8 (±9.3) years, and 424 (65.1%) of them were married. In terms of educational status, 333 (51.2%) of them had a bachelor’s degree (BSc/BED) or higher education, and 248 (38.1%) of them had been working for 16 or more years. In Ethiopia, there is no national minimum wage. The minimum wage exists only for public sector workers which are around 420 ETB (56). In this study, 280 (43%) respondents had a monthly salary of less than 6,000 ETB, and 27 (4.1%) of them had a family size of seven or more (Table 1).

**Table 1:** Socio-demographic characteristics of primary school teachers in Gimbi Town, western Ethiopia, 2021 (*N* = 651).

Variables	Frequency (n)	Percentage (%)
**Sex**		
Male	389	59.8
Female	262	40.2
**Age**		
21–29 years	159	24.4
30–39 years	311	47.8
40–49 years	116	17.8
≥50 years	65	10.0
**Marital status**		
Single	157	24.1
Married	424	65.1
Divorced/widowed	70	10.8
**Educational status**		
Teacher training institute (TTI) certificate	22	3.3
Diploma	296	45.5
Degree and above	333	51.2
**Monthly salary in ETB**		
4,500–6,000	280	43.0
6,000–8,499	155	23.8
8,500–10,500	164	25.2
>10,501	52	8.0
**Teaching experience**		
1–5 years	97	14.9
6–10 years	210	32.3
11–15 years	96	14.7
16–22 years	248	38.1
**Family size**		
≤3	428	65.7
4–6	196	30.1
≥7	27	4.1
**Family support**		
Yes	320	49.2
No	331	50.8

ETB, Ethiopian Birr (currency).

### Behavioral and sleep characteristics of the respondents

Of the study participants, only 105 (16.1%) of them reported they were performing physical exercise at least two times per week. Regarding substance use, 55 (8.4%) of the teachers were alcohol drinkers, 39 (6.0%) were khat chewers, and 47 (7.2%) were cigarette smokers. Fifty (7.7%) respondents reported sleeping less than the recommended amount of sleep per night (<7 hours per day) (Table 2).

**Table 2:** Behavioral and sleep characteristics of primary school teachers in Gimbi town, western Ethiopia, 2021 (*N* = 651).

Variables	Frequency (*n*)	Percentage (%)
**Physical exercise**		
Yes	105	16.1
No	546	83.9
**Alcohol drinking**		
Yes	55	8.4
No	596	91.6
**Khat chewing**		
Yes	39	6.0
No	612	94.0
**Cigarette smoking**		
Yes	47	7.2
No	604	92.8
**Sleeping hours per day**		
<7 h/day	50	7.7
≥7 h/day	601	92.3
**Sleep disturbance**		
Yes	254	39.0
No	397	61.0

### Work-related factors of study participants

Nearly half, 328 (50.4%) of the teachers reported that they had high job demand, while, 382 (58.7%) of them reported having low control over their jobs. Three hundred twenty-seven (50.2%) of participants had high management support for their work, whereas 331 (50.8%) had low peer support. In terms of participant roles, 351 (53.9%) of the respondents reported role ambiguity in their schools. Concerning participants’ relationships with their coworkers in the work area, more than two-thirds, 450 (69.1%) of them had reported poor staff relationships. A majority, 392 (60.2%) of participants had poor perceptions of their physical school environment and 272 (41.8) of them had high-risk perceptions of the COVID-19 virus. Although the Ethiopian Ministry of Education limited the class size to 25 students per classroom during the COVID-19 pandemic, the majority, 541 (83.1%) of the teachers reported that there were more than 25 students in the classroom. The majority, 365 (56.1%) teachers stated that most students were misbehaving, and 291 (44.7%) of them were reported as dissatisfied with their jobs (Table 3).

**Table 3:** Work-related factors of primary school teachers in Gimbi town, western Ethiopia, 2021 (*N* = 651).

Variables	Frequency (*n*)	Percentage (%)
**Job demand**		
High	328	50.4
Low	323	49.6
**Job control**		
High	269	41.3
Low	382	58.7
**Management support**		
High	327	50.2
Low	324	49.8
**Peer support**		
High	320	49.2
Low	331	50.8
**Role ambiguity**		
High	351	53.9
Low	300	46.1
**Change management**		
High	379	58.2
Low	272	41.8
**Relationship**		
Good	201	30.9
Poor	450	69.1
**Job change**		
Good	289	44.4
Poor	362	55.6
**Perception of physical work environment**		
Good	269	39.8
Poor	392	60.2
**Perceived job satisfaction**		
Satisfied	360	55.3
Dissatisfied	291	44.7
**Number of students per class**		
≤25 students	110	16.9
>25 students	541	83.1
**Students discipline**		
Good	286	43.9
Bad	365	56.1
**Risk perception of COVID-19 virus**
High	272	41.8
Low	379	58.2

### Prevalence of occupational stress

The finding of this study revealed that the overall prevalence of OS in the past 4 months was 50.1% (*n* = 326) [95% CI (46.1, 53.9)]. This implies that one out of every two school teachers experienced OS during the second wave of COVID-19.

### Factors associated with occupational stress

Bivariable and multivariable analysis was done to see factors associated with OS. Accordingly, age, educational status, job satisfaction, physical work environment, student discipline, relationship on the job, management support, peer support, and risk perception of the COVID-19 virus were found to be the independent variables associated with OS in the bivariable binary logistic regression analysis at a value of *p* of <0.2. However, after controlling for confounding variables in the multivariable binary logistic regression analysis, job satisfaction and risk perception of COVID-19 infection remained to be significant factors determining the occurrences of OS at a value of *p* of <0.05.

Teachers who were dissatisfied with their work were about two times more likely to develop OS compared to teachers who were satisfied with their work [AOR: 2.06, 95% CI (1.43–2.97)] at a value of p of value of *p* ≤ 0.001. Moreover, the odds of having OS were 2.20 times higher among teachers who had a high-risk perception of COVID-19 infection than among those who had a low-risk perception of it [AOR: 2.20; 95% CI (1.46–3.31)] at a value of *p* of ≤0.001 (Table 4).

**Table 4:** Binary logistic regression analysis of factors associated with occupational stress among primary school teachers in Gimbi town, Oromia, Ethiopia 2021 (*N* = 651).

Variables	Occupational Stress		COR with 95% CI	AOR with 95% CI	value of *p*
	Yes (326)	No (325)			
	*n* (%)	*n* (%)			
**Age in years**					
21–29	78 (49.1)	81 (50.9)	1.27 (0.71–2.28)	1.22(0.96–5.14)	0.262
30–39	154 (49.5)	157 (50.5)	1.29 (0.76–2.22)	1.62(0.87–5.63)	0.130
40–49	66 (56.9)	50 (43.1)	1.74 (1.55–3.22)	1.46 (0.89–4.16)	0.143
≥50	28 (43.1)	37 (56.9)	1	1	
**Educational status**					
Certificate of TTI	14 (63.6)	8 (36.4)	1.80 (0.74–4.41)	1.13(0.40–3.24)	0.815
Diploma	148 (50.0)	148 (50.0)	1.03 (0.75–1.41)	1.16(0.80–1.70)	0.405
Degree and above	164 (49.2)	169 (50.8)	1	1	
**Job satisfaction**					
Dissatisfied	179 (61.5)	112 (38.5)	2.32 (1.69–3.18)	**2.06 (1.43–2.97)**	0.001*
Satisfied	147 (40.8)	213 (59.2)	1	1	
**Physical work environment**					
Good	208 (53.1)	184 (46.9)	1	1	
Poor	118 (45.6)	141 (54.4)	1.35(0.99–1.85)	1.003 (0.70–1.44)	0.986
**Student discipline**					
Poor	199 (54.5)	166 (45.5)	1	1	
Good	127 (44.4)	159 (55.6)	1.10(1.79–2.05)	0.78(0.55–1.10)	0.152
**Relationship on job**					
Poor	214 (47.6)	236 (52.4)	1.38(0.99–1.93)	0.19 (1.02–0.89)	0.083
Good	112 (55.7)	89 (44.3)	1	1	
**Management support**					
Low	175 (54.0)	149 (46.0)	1.37(1.01–1.86)	0.70 (0.46–1.04)	0.077
High	151 (46.2)	176 (53.8)	1	1	
**Peer support**					
Low	184 (55.6)	147 (44.4)	1.57(1.15–2.14)	1.23 (0.82–1.86)	0.317
High	142 (44.4)	178 (55.6)	1	1	
**Risk perception of the COVID-19 virus**					
High	168 (61.8)	104 (38.2)	2.26 (1.64–3.11)	**2.20 (1.46–3.31)**	0.000**
Low	158 (41.7)	221 (58.3)	1	1	

1, reference category; AOR, adjusted odds ratio; CI, confidence interval; COR, crudes odds ratio; COVID-19, Coronavirus disease 19; *, significant at value of *p* < 0.05, **Significant at value of *p* < 0.001 in multivariable logistic regression analysis, Model fitness: Hosmer and Lemeshow test was 0.352 (value of *p* > 0.05). The values in are the values of the adjusted odds ratio (AOR) of the significant factors in the multivariable logistic regression.

## Discussion

Occupational stress is becoming an alarmingly growing public health concern in educational sectors specifically in teachers globally, and the problem worsened during the COVID-19 pandemic (57, 58). Understanding the extent and causes of the problem plays a critical role in promoting teachers’ mental health and teaching quality. The current study aimed to examine the prevalence and factors influencing OS among primary school teachers during the second wave of the COVID-19 pandemic in Gimbi town, Western Ethiopia. In the current study, the prevalence of OS during the Covid-19 pandemic was found to be 50.1% (*n* = 326), 95% CI: [46.1–53. 9]. The fact that primary school teachers in Ethiopia work in substandard schools with limited resources, are overburdened, and have hectic schedules, all of which exacerbate the occurrences of OS.

The proportion of male participants in this study was higher (59.8%) than that of females. This may be because females were more likely than males to dread the COVID-19 pandemic (59) and that females felt more responsible for taking care of their vulnerable family members, such as children and elderly parents, which led them to stay away from work (school) during the data collection period than male participants. Another possible reason could be that in Ethiopia, females are underrepresented in all areas, including education, employment, politics, and other important decision-making. Males have the most authority, while females are generally considered subordinate to their husbands and fathers (60, 61). Therefore, in Gimbi town there could be a greater number of male than female teachers, which results from a higher proportion of male participants than females.

The prevalence of OS in the current was higher than studies conducted in Libya (39.5%) (62), Nigeria (38.2%) (63), South Africa (31.1%) (23), Pakistan (44.9%) (21), Greece (42%) (18), and Kosovo 33.2% (19). The probable reason for this variation could be differences in measurement tools used (Libya: DASS-21, Nigeria: OS-24 items, South Africa: Fimian Teacher Stress Inventory, and Kosovo-NSAD); workplace environment, time difference, study setting, and differences in the study population, living standards, and economic fluctuation. In comparison to studies in India (65%) (20), Chile (86%) (9), Egypt (100%) (24), and another study in Ethiopia (58.2%) (25), our finding was lower in a proportion of teachers having OS. This variation could be attributed to differences in local characteristics such as perceptions, traditions, study tools, and living standards, which could have compounded or mitigated the effects of OS (64).

The results of the current study indicated that teachers who were dissatisfied with their jobs were more likely to have OS. This result was supported by studies conducted in Ghana (65) and Columbia (66). The possible reason could be that job dissatisfaction results from teachers’ high psychological demand and their low control over it (67) which then exposes them to frequent psychological loads that increase stress and other health problems (68). Although job dissatisfaction can result in discomfort, low self-esteem, sadness, and devaluation, all of which contribute to high levels of stress at work (69, 70). The other possible explanation could be that teachers are expected to provide academic instruction, social–emotional support, and build relationships with students and families while often receiving inadequate compensation or support from administration and leadership, which can lead them to dissatisfaction and result in stress development (71). Furthermore, dissatisfied teachers during the COVID-19 pandemic experienced unprecedented and abrupt changes to their working conditions. Schools were ordered to close with immediate effect on 12th March 2020, and proposed reopening timelines were extended unpredictably as the pandemic evolved (72, 73). Therefore, dissatisfied teachers experienced more OS as compared to satisfied individuals.

Risk perception is the prime factor that elicits psychological and behavioral responses in people during public crisis events, and it has a significant impact on both daily life decisions and behaviors (74). Our analysis confirmed that the high-risk perception of the COVID-19 pandemic increased the development of OS. This finding is in concordance with other research reports (75–77). This could be because high perceived risk puts people in a distressed and anxious state, which in turn motivates them to engage in problem-solving activities to resolve it (78). A study has also revealed that people with a higher perception of the risk of the COVID-19 pandemic are more likely to panic and respond unfavorably (77). As a result, the higher the level of risk perception, the greater the psychological stress people will develop. Another plausible reason could be that teachers who perceived high risk for the COVID-19 pandemic were able to perceive the stressful situations induced by the COVID-19 pandemic, mainly pandemic fear, organizational pandemic response, and job insecurity (79–81). Pandemic fear refers to an individual’s perceived risks of being infected by COVID-19 and other health consequences (e.g., a high mortality rate). Job insecurity reflects individual concerns about the prospect of job continuity due to the economic downturn triggered by the pandemic. The organizational response to the pandemic represents whether organizational measures are adequate to protect every worker from the pandemic.

## Strength and limitations of the study

This study was conducted to examine the perceived OS and associated factors among primary school teachers in western Ethiopia, who are more likely to suffer from the problem, particularly during the COVID-19 pandemic. There are only a few published studies in the scientific literature that examine the prevalence and risk factors of OS among school teachers, specifically in the context of the COVID-19 pandemic. The study used a standardized tool to assess the variables and contributes evidence from a relatively large sample with very good response rates (96.8%). This study would likely add a significant amount of evidence to the scant existing literature on the prevalence and causes of OS during the COVID-19 pandemic. However, some limitations should be considered when interpreting our findings. First, due to the nature of the study design, it was not possible to establish a clear temporal relationship between significantly associated factors and occupational stress. Second, even if we have used the four-month prevalence report, the results of this study might be under or over-reported due to recall bias. Moreover, participants’ responses may also be susceptible to social desirability bias, which leads them to give socially acceptable answers. To decrease social desirability, however, precautions were taken by making sure that only study participants were present during data collection and that data confidentiality was upheld. Despite these limitations, we believe that the study provided a reasonably accurate assessment of OS and associated risk factors among primary school teachers in Gimbi town, western Ethiopia.

## Conclusion

The survey of this study disclosed a high prevalence of occupational stress among primary school teachers during the second wave of COVID-19. Job dissatisfaction and a high-risk perception of COVID-19 infection were significant and independent predictors of the occurrence of occupational stress in school teachers. Enhancing stress management skills and focusing on primary prevention of identified risk factors was recommended to curtail the condition.

## Data availability statement

The original contributions presented in the study are included in the article/supplementary material, further inquiries can be directed to the corresponding author.

## Ethics statement

The studies involving human participants were reviewed and approved by Institutional Ethical Review Board (IRB) of the University of Gondar. The patients/participants provided their written informed consent to participate in this study.

## Author contributions

AT, KA, GK, and TA conceptualized and drafted the proposal, supervised the data collection, and cleaned the data. AT involved in data analysis, presented the results and discussions, wrote up the draft manuscript, and reviewed and finalized the manuscript document. KA wrote up the research proposal, analyzed the data, presented the results and discussions, and wrote up the draft manuscript. GK involved in data analysis, participated in the presentation and interpretation of results and discussions, and reviewed the draft manuscript document. TA involved in data analysis, participated in the presentation and interpretation of results and discussions, and reviewed the draft manuscript document. All authors contributed to the article and approved the submitted version.

## Conflict of interest

The authors declare that the research was conducted in the absence of any commercial or financial relationships that could be construed as a potential conflict of interest.

## Publisher’s note

All claims expressed in this article are solely those of the authors and do not necessarily represent those of their affiliated organizations, or those of the publisher, the editors and the reviewers. Any product that may be evaluated in this article, or claim that may be made by its manufacturer, is not guaranteed or endorsed by the publisher.

## Abbreviations

AOR: Adjusted odds ratio
BSC/BED: Bachelors of Science or Bachelors of Education
CI: Confidence interval
COR: Crude odds ratio
ETB: Ethiopian Birr
HSE: Health and safety executive
OS: Occupational stress
TOSS: Teachers occupational stress scale
UK: United Kingdom
